# Transcatheter aortic valve implantation: first choice for aortic stenosis?

**DOI:** 10.1007/s12471-020-01419-9

**Published:** 2020-04-15

**Authors:** P. den Heijer

**Affiliations:** Heart Centre, Amphia Hospital, Breda, The Netherlands

**Fig. 1:**
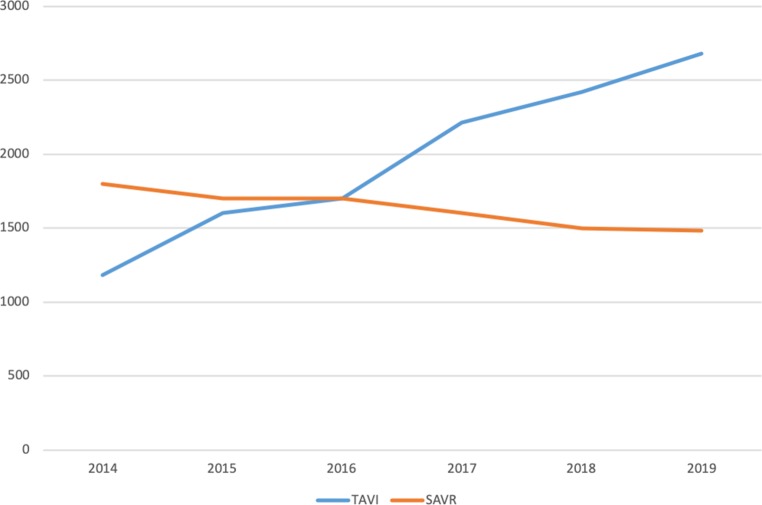
Estimated numbers of transcatheter aortic valve implantation (*TAVI*) and surgical aortic valve replacement (*SAVR*) in the Netherlands, 2014–2019

Fifteen years after the first transcatheter aortic valve implantation (TAVI) was performed in the Netherlands, it is time to consider whether TAVI should be considered the first-choice treatment for patients with acquired calcified aortic valve stenosis (AS). In sheer procedure numbers, it is. In the Netherlands, the lines of surgical aortic valve replacement (SAVR) and TAVI crossed in 2016 (Fig. 1). In this special edition of the *Netherlands Heart Journal*, in the position statement by the Dutch Working Group of Transcatheter Heart Interventions, De Jaegere et al. argue that the heart teams in TAVI centres, as the gatekeepers of treatment decisions, should have the final word in advising our patients between conservative management of AS, SAVR and TAVI [1]. Hence the question is: should heart teams consider TAVI to be the treatment of first choice *in all patients*? Should they opt for TAVI even in younger patients, without co-morbidity and therefore with a low operative risk?

There are several angles from which to approach this question. There is also a stark difference in opinions. If asked, any interventional cardiologist will say, without hesitation: yes, TAVI is the best intervention for all patients who need an aortic valve bioprosthesis. In 2019, two breakthrough trials proved the non-inferiority or superiority of TAVI compared to SAVR, for several relevant hard clinical endpoints, such as death, disabling stroke, rehospitalisation and new-onset atrial fibrillation [2, 3]. As expected, these publications have met with much less undiluted euphoria in the cardiothoracic surgeon’s community. Cardiac surgeons point to higher rates of paravalvular leakage and new permanent pacemaker implant rates with TAVI. Although the former has been largely resolved with current-generation TAVI systems and is perhaps compensated by lower gradients, the latter remains of concern, especially for younger patients with long life expectancies. Even more important, such relatively young patients form the focus of a much-used cardiac surgeon’s argument: durability of the bioprosthesis. In the past, there has been less debate over surgical bioprostheses with sometimes questionable rates of degeneration, leading to frequent valve-in-valve TAVI procedures [4]. Moreover, durability of TAVI bioprostheses is subject to unprecedented scrutiny and scientific research [5]. Still, the dispute concerning uncertain TAVI valve failure rates beyond 5 years, as compared to some of the best surgical valves [5], remains a valid argument and true concern. Perhaps in the near future, patient age and life expectancy will become more important arguments in our heart teams than surgical risk.

Physicians and heart teams are bound by guidelines. Although the current AHA/ACC and ESC/EACTS guidelines allow TAVI for patients with intermediate surgical risk, they do not for young patients with low operative risks [6, 7]. As a result of the recent low-risk studies, future updates of these guidelines may be less restrictive for TAVI. Importantly, the 2017 ESC/EACTS guidelines consider not only surgical risk, but have also added anatomical suitability for TAVI to the decision tree. Patients with a low risk for SAVR can be at high risk for suboptimal outcome with TAVI, for instance when pathology consists of a heavily calcified bicuspid aortic valve stenosis.

Another, increasingly relevant viewpoint in this discussion is patient preference [8]. Most patients, if given a choice, prefer less invasive therapy over open-heart surgery. Finally, health authorities, largely driven by the high cost of TAVI valves, have an influence on medical practice and heart team decision-making. The Dutch National Health Care Institute (*Zorginstituut Nederland*) takes a highly restrictive view. This institute regards SAVR as the standard therapy for calcified aortic stenosis, striving to allow TAVI, as insured health care, only for inoperable patients and patients with a high surgical risk. The Dutch Working Group of Transcatheter Heart Interventions has been tasked to implement these regulations in the heart teams of the Dutch TAVI centres. This work in progress is a difficult task, seeking a compromise between all angles and opinions as expressed in this editorial comment, and at the same time following the international guidelines.

Fifteen years after the first TAVI was performed in the Netherlands, this elegant and minimally invasive therapy can be considered first choice only in selected patients. However, although there will always be a place for SAVR, we can hope for, and indeed expect in the coming years, an ongoing shift toward opting for TAVI in our heart teams.
